# Effects of CTC_P_ Modification on Microstructure and Wear Behavior of CTC_P_-NiCrBSi/Heat Resistant Steel Composite Layer

**DOI:** 10.3390/ma11112202

**Published:** 2018-11-07

**Authors:** Jianjun Zhang, Shuzeng Hou

**Affiliations:** 1 Key Laboratory of Fluid and Power Machinery, Ministry of Education, School of Materials Science and Engineering, Xihua University, Chengdu 610039, China; 2 Material Corrosion and Protection Key Laboratory of Sichuan Province, Sichuan University of Science & Engineering, Zigong 643000, China; 3 College of Mechanical Engineering, Sichuan University of Science & Engineering, Zigong 643000, China

**Keywords:** composite layer, cast tungsten carbide, dissolution, elevated temperature abrasion

## Abstract

A CTC_P_-NiCrBSi/heat resistant steel composite layer was designed and fabricated by vacuum fusion sintering. The structure of the composite layer was similar to reinforced concrete. Numerous reinforced regions with a cylindrical shape were evenly distributed in the heat resistant steel. Modified cast tungsten carbide particles (CTC_P_) reinforced NiCrBSi matrix composite constituted the reinforced region (CTC_P_-NiCrBSi). The microstructure of the composite layers was investigated by scanning electron microscope (SEM), energy dispersive x-ray spectrometer (EDS), and image analysis. The wear behavior of the composite layer was estimated on the ring-on-disc rig at a temperature range of room temperature (RT) to 800 °C in air. The microstructure and wear behavior of the composite layer with modified CTC_P_ were compared with those with primary CTC_P_. The results showed that the poor chemical resistance of W_2_C and the interdiffusion of elements were responsible for the dissolution of unmodified CTC_P_ in the molten NiCrBSi alloy. A WC outer shell formed on the surface of the CTC_P_ after surface carburizing modification. The WC outer shell could effectively resist the dissolution of CTC_P_ in NiCrBSi during the sintering process. The content of WC/W_2_C in modified CTC_P_-NiCrBSi increased by about 12.0 vol. % when compared with that in the primary CTC_P_-NiCrBSi. The wear rate of the composite layer with modified CTC_P_ was lower than that with primary CTC_P_ between RT and 700 °C. The wear rates of the composite layer decreased with increasing temperature from RT to 700 °C and increased above 700 °C.

## 1. Introduction

Crushing, compacting, classifying, or delivering of hot minerals often lead to the severe wear of the structural parts of machines [1]. Hard-particle reinforced metal matrix composite (HPRMMC) is widely used to increase the lifetime of machinery equipment exposed to severe wear conditions [2,3,4,5]. The HPRMMC layer is the application of a hard, wear resistant HPRMMC to the surface of a softer metal substrate performed by surface technologies like laser cladding, surface overlaying, and fusion sintering. The HPRMMC layer usually has a whole layer structure. It is an effective method to reduce wear caused by abrasion or impact. Embedded particles increase hardness and improve the abrasion resistance of the layers, while seriously deteriorating the ductility and impact resistance of the layers. Moreover, the residual stress resulting from the mismatch in the thermal expansion coefficients and Young’s modulus between the layer and the substrate reduces the fracture toughness of the bimaterial. Therefore, the layers often crack and even peel off along the joint interface during preparation and application [6,7,8]. These disadvantages have restricted the extensive application of HPRMMC layers in industry. Traditional methods for improving the toughness of the HPRMMC layers rely on controlling parameters such as size, volume fraction, and the distribution of particles as well as the properties of the matrix. So far, it has not provided the necessary level of toughness enhancement while retaining the abrasion resistance of the layers [9]. Published experimental results indicate that local particle reinforcement distribution patterns can play a very significant role in controlling HPRMMC properties as well as damage tolerance [10,11,12]. This method may provide a reference for the toughness improvement of HPRMMC layers.

HPRMMC layers consisting of cast tungsten carbide particles (CTC_P_) and NiCrBSi matrix (CTC_P_-NiCrBSi) are commonly employed. In the group of tungsten carbides, tungsten monocarbide (WC) and cast tungsten carbide (CTC) are used industrially. CTC is a eutectic mixture of WC and W_2_C (WC/W_2_C). CTC exhibits distinctly higher hardness and toughness when compared with WC. A major disadvantage of CTC lies in the fact that during the deposition of layers, the CTC_P_ are partly or even completely dissolved by the molten NiCrBSi matrix. The dissolution inevitably influences the wear resistance of the CTC_P_-NiCrBSi composite layers. In order to reduce or eliminate the dissolution of CTC_P_ during deposition, a patent process has been employed which modifies the CTC_P_ by carburizing [13]. Jones et al. studied the effects of CTC_P_ modification on the erosion–corrosion properties of the CTC_P_-NiCrBSi composite layer. The results showed that the rate of the modified CTC_P_-NiCrBSi composite layer was approximately a third of the rate of the unmodified CTC_P_-NiCrBSi composite layer [14]. However, few publications have reported on the effects of CTC_P_ modification on the wear behavior of the CTC_P_-NiCrBSi composite layer.

**Figure 1:**
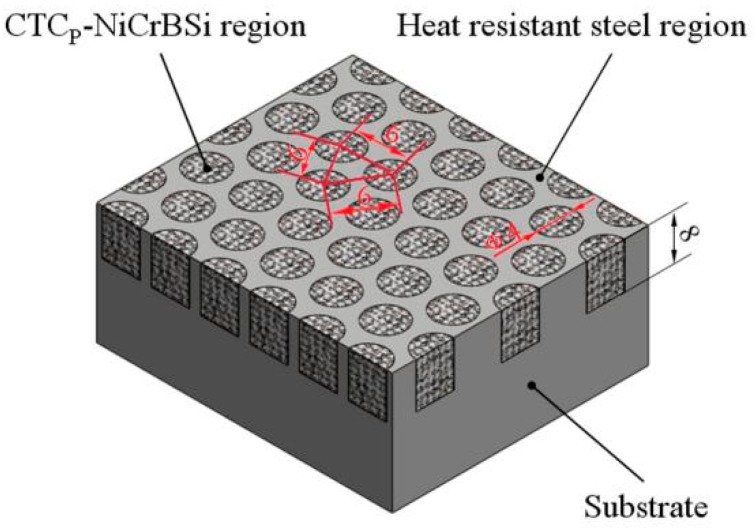
Schematic diagram of the CTC_P_-NiCrBSi/heat resistant steel composite layer (Dimensions are in mm).

In the present work, a CTC_P_-NiCrBSi/heat resistant steel composite layer similar to concrete in architecture was designed and fabricated by vacuum fusion sintering. Figure 1 presents the schematic diagram of the composite layer. The CTC_P_-NiCrBSi/heat resistant steel composite layer was composed of many discrete circular reinforced regions distributed uniformly in the heat resistant steel region. The discrete reinforced region was comprised of the CTC_P_-NiCrBSi composite. The continuous heat resistant steel region extended down into substrate. In this architectural structure, the discrete CTC_P_-NiCrBSi regions could better strengthen the layer and the softer continuous heat resistant steel region could better toughen the layer, simultaneously. The effects of CTC_P_ modification on the microstructures and wear behaviors of the CTC_P_-NiCrBSi/heat resistant steel composite layers are carried out below.

## 2. Materials and Experimental Procedure

### 2.1. Raw Materials

Heat resistant steel with a composition of 0.45 wt. % C, 23.50 wt. % Cr, 19.21 wt. % Ni, 1.58 wt. % Si, 1.24 wt. % Mn, and balance Fe was used as the substrate. The matrix of the reinforced regions used was self-fluxing NiCrBSi alloy powder with a size less than 25 μm, whose chemical composition was 0.06 wt. % C, 6.52 wt. % Cr, 3.28 wt. % B, 4.62 wt. % Si, 2.71 wt. % Fe, and Ni in balance. The solidus temperature of the NiCrBSi alloy was 980 °C, and its liquidus temperature was 1050 °C. Typical crushed CTC_P_ with sizes ranging from 40 to 100 μm were used as reinforcement. Some of the CTC_P_ were modified by the carburizing process that involved mixing CTC_P_ with carbon black and heating the mixture at 1550 °C for 90 min in vacuum.

### 2.2. Fabrication of the Composite Layer

The heat resistant steel substrate with blind holes was machined according to a predetermined design. In the surface layer of the substrate, there were ordered blind holes of 4 mm in diameter, 8 mm in depth and with a fix distance of 6 mm between the holes. Meanwhile, the NiCrBSi alloy powder with 40 vol. % CTC_P_ (unmodified or modified) was mixed for 4 h in a tumbling mixer. Then, the powder mixture was filled in the holes of the substrate and compacted under pressure. Finally, the assembled specimen was put in the vacuum furnace and sintered according to the heating process as follows. First, the specimen was heated to 900 °C with a heating rate of 10 °C/min. Second, the temperature was held for about 30 min to reduce the temperature gradient of the specimen. Third, heating was resumed to the sintered temperature of 1100 °C with a heating rate of 5 °C/min. Finally, the specimen was processed at 1100 °C for 20 min followed by slow cooling within 8 h to room temperature. From Figure 1, it can be seen that the CTC_P_-NiCrBSi regions occupied about 40 vol. % of the CTC_P_-NiCrBSi/heat resistant steel composite layer.

### 2.3. Microstructure Characterization

The microstructures of the CTC_P_-NiCrBSi regions were observed by means of scanning electron microscope (SEM). Chemical compositions of the phases and elemental distributions of the interfaces were analyzed by utilizing an energy dispersive x-ray spectrometer (EDS, TESCAN, Brno, Czech).

Quantitative image analysis was carried out to measure the area fractions of the microstructure constituents (such as dissolution zone, residual WC/W_2_C, WC shell, and precipitates) in the CTC_P_-NiCrBSi regions. Surface areas used for image analysis were randomly chosen, and more than 30 CTC_P_ were included in the selected area to provide statistically meaningful data. The magnification of the images was 500× and the measuring field was 450 × 340 μm in size. Each constituent analysis was repeated four times and the results were averaged. The constituent volume fraction was considered equal to the constituent area fraction determined by image analysis.

To determine the microhardness of different microstructure constituents, HV_0.05_ (Vickers microhardness tester using a 50-g load for 10 s) was used. The hardness tester was equipped with an optical microscope to allow the user to evaluate in which microstructure constituent the measurement was conducted. The measurement of the hardness values was conducted automatically, but sometimes needed manual adjustment if the indention was not properly detected. At least 16 measurements were conducted on each microstructure constituent at random positions, providing the microhardness values of the different microstructure constituents.

### 2.4. Abrasion Test

**Figure 2:**
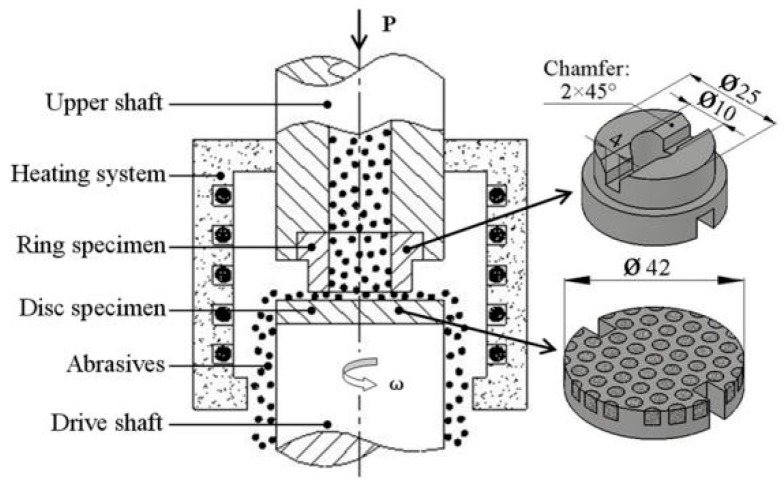
Schematic diagram of the abrasion test rig and the specimens (Dimensions are in mm).

Abrasion tests were conducted in a ring-on-disc rig with interfacial abrasive particles at a temperature range of room temperature (RT) to 800 °C in air. The schematic diagram of the abrasion test rig and the specimens are shown in Figure 2.

The ring material used in this study was high Cr white cast iron with a hardness of HRC55. The disc specimens were made from the CTC_P_-NiCrBSi/heat resistant steel composite layers. Angular quartz sand (HV980~1100) [15] with a grain size between 180 and 380 μm was used as the abrasive. The ring and disc rotated with a relative speed of 50 rpm (giving a linear speed of 0.04579 m/s) under a load of 20 N. After a wear distance of 82.422 m, the tests were stopped and the mass losses of the disc were measured using an electronic balance with an accuracy of 0.0001 g. From the mass loss of the disc and the length of wear path, wear rate *W_a_* was derived according to the following equation:

(1) $$W_{a}=\frac{\Delta m}{\left\{\left[\rho_{p}\alpha +\rho_{b}\left(1-\alpha \right)\right]\beta +\rho_{m}\left(1-\beta \right)\right\}\times l}$$

where 𝜟*m* represents the mass loss of the disc; *ρ_p_*, *ρ_b_*, and *ρ_m_* represent the densities of CTC_P_, NiCrBSi, and heat resistant steel, respectively; *α* and *β* represent the volume fraction of CTC_P_ in the CTC_P_-NiCrBSi region and CTC_P_-NiCrBSi regions in the composite layer, respectively; and *l* represents the length of the wear path. Each abrasion test was repeated three times and the wear rate value was averaged.

After the wear tests, the worn surfaces and cross sections of the discs were examined using SEM and EDS.

## 3. Results and Discussion

### 3.1. Microstructure of the CTC_P_-NiCrBSi Region

**Figure 3:**
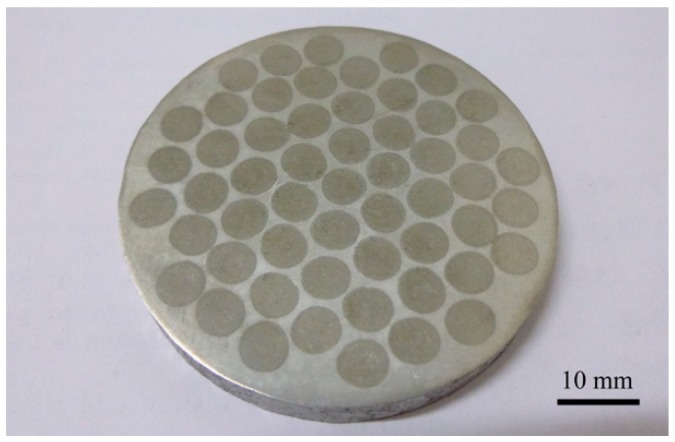
Macrograph of the CTC_P_-NiCrBSi/heat resistant steel composite layer fabricated.

Shrinkage of the powder mixture in the holes led to a wavy surface of the CTC_P_-NiCrBSi/heat resistant steel composite layer during fusion sintering. Figure 3 shows the macrograph of the CTC_P_-NiCrBSi/heat resistant steel composite layer fabricated in this study after the wavy surface was planned. It can be seen that the CTC_P_-NiCrBSi/heat resistant steel composite layer had no obvious defects such as cracks and pores.

**Figure 4:**
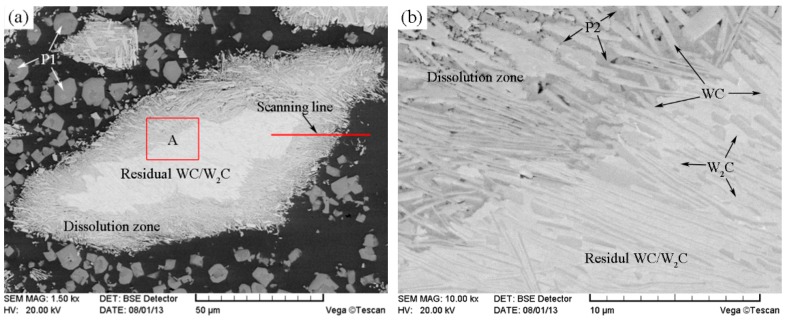
(**a**) SEM image of the unmodified CTC_P_-NiCrBSi, and (**b**) magnified SEM image of the zone “A” in (**a**).

**Figure 5:**
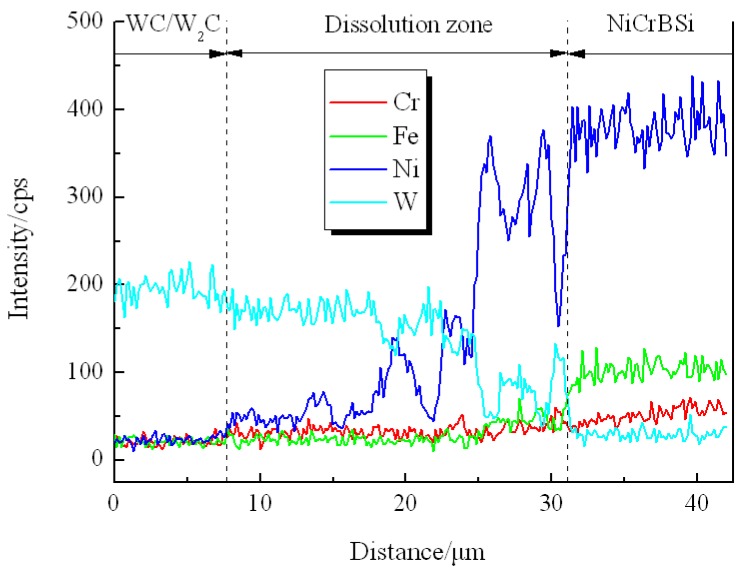
EDS line scan from unmodified CTC_P_ to NiCrBSi.

Figure 4 shows the SEM images of the unmodified CTC_P_-NiCrBSi region in the unmodified CTC_P_-NiCrBSi/heat resistant steel composite layer. It is evident that the unmodified CTC_P_ displayed considerable dissolution and a small amount of primary CTC was left in the core. A considerable number of blocky precipitates (marked as P1) away from the CTC were formed in the NiCrBSi matrix (Figure 4a). EDS spot analysis confirmed that they were (W, Ni, Cr)-rich carbides. The magnified image shows more details of the CTC and the dissolution zone (Figure 4b). The lamellar dual-phase structure of the CTC can be clearly distinguished where the brighter phase containing approximately 66 at. % W was W_2_C; and the slightly darker phase containing approximately 50 at. % W was WC. In the dissolution zone, the slightly darker strip phase containing approximately 50 at. % W was WC, which should be the residual phase from the CTC; the darker phase (marked as P2) was (W, Ni)-rich carbide, which should be the reaction product of W_2_C with a NiCrBSi matrix. The result of the EDS line scan through the dissolution zone is shown in Figure 5 (scanning line see Figure 4a). It can clearly be seen that in the dissolution zone, the Ni, Fe, and Cr contents increased and the W content decreased from CTC to the NiCrBSi matrix and the gradient of concentration was gradual, which indicates that strong elemental diffusion occurred in the dissolution zone.

**Figure 6:**
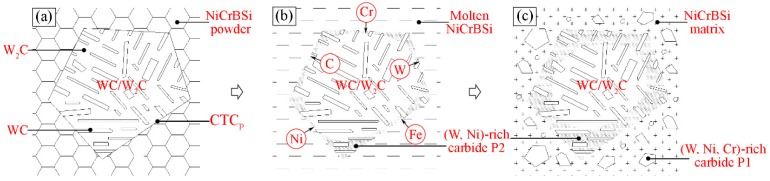
Dissolution schematic diagram of the unmodified CTC_P_ in NiCrBSi alloy: (**a**) Before sintering; (**b**) Interdiffusion of elements and (W, Ni)-rich carbide forming; (**c**) (W, Ni, Cr)-rich carbide precipitating.

In the following, we would like to explain the dissolving behavior of CTC_P_ in the NiCrBSi matrix. Figure 6 describes the dissolution mechanism according to the above results. Before vacuum fusion sintering, the CTC_P_ was surrounded by NiCrBSi alloy powder (Figure 6a). During the sintering, the NiCrBSi alloy powder became a liquid phase because of its low melting point, and element interdiffusion occurred between molten NiCrBSi and CTC_P_. Elements such as Ni, Cr, and Fe diffused from the molten NiCrBSi to CTC_P_ while the W and C diffused in the opposite direction. In the diffusion process, the W_2_C phase in CTC_P_ reacted with Ni, Cr, and other elements diffusing from the molten matrix to form (W, Ni)-rich carbide, while the WC phase remained mostly intact (Figure 6b). Elements such as Ni, Cr, and Fe in molten NiCrBSi reacted with W and C diffusing from CTC_P_ to form (W, Ni, Cr)-rich carbides, which were precipitated from the melt during subsequent cooling (Figure 6c). This diffusion reaction is similar to the work of Molina [16].

**Figure 7:**
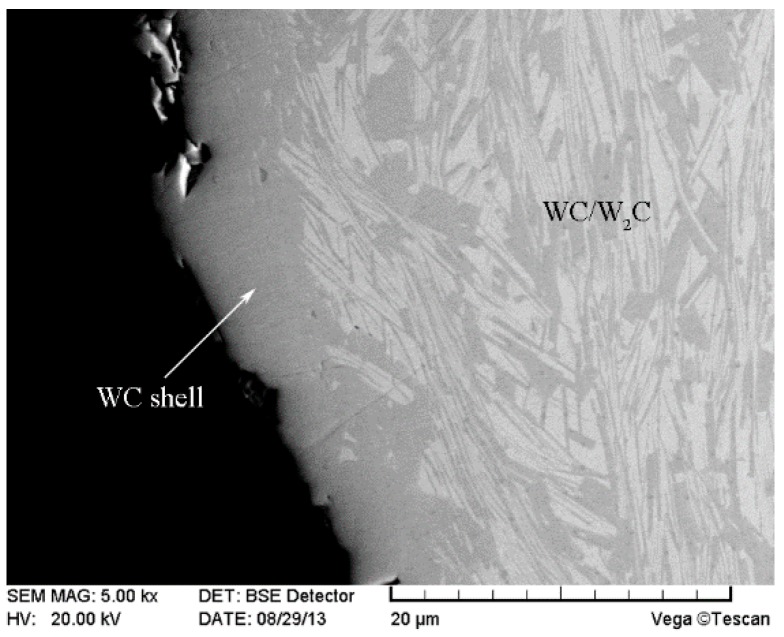
SEM image of the modified CTC_P_.

Based on the above analysis, the poor chemical resistance of the W_2_C phase is responsible for the dissolution of CTC_P_. The WC phase has a higher chemical resistance than the W_2_C phase. This suggests that CTC_P_ could be modified by carburizing to produce an outer shell of WC to prevent or reduce dissolution during the deposition. Figure 7 shows the SEM image of the modified CTC_P_ according to the carburizing process mentioned previously. It can be seen that the particle had a core of WC/W_2_C eutectic surrounded by a slightly darker shell approximately 8 μm in thickness. EDS analysis confirmed that the shell was WC. During the carburizing, the carbon black reacted with the W_2_C in the CTC, and the W_2_C was converted to WC which could no longer be distinguished from the original WC phase. Thus, a dense WC shell formed on the CTC_P_ surface.

**Figure 8:**
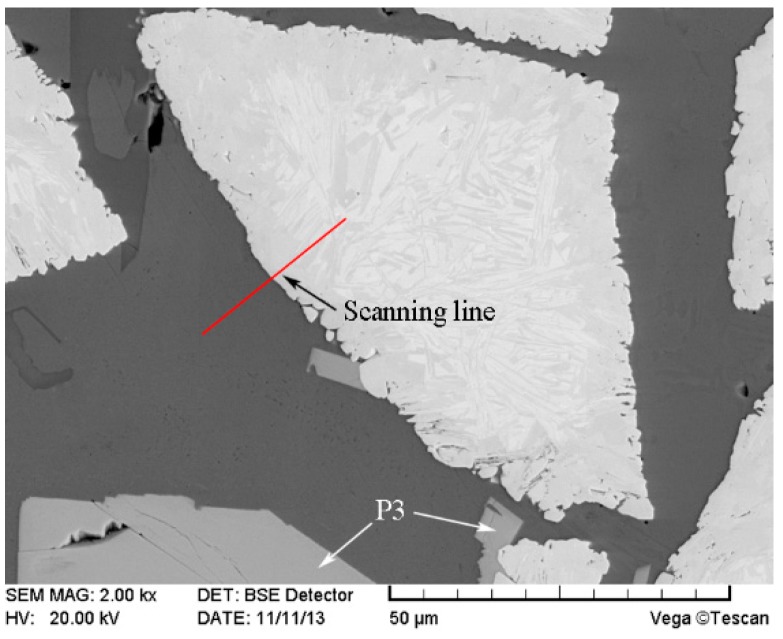
SEM image of the modified CTC_P_-NiCrBSi.

**Figure 9:**
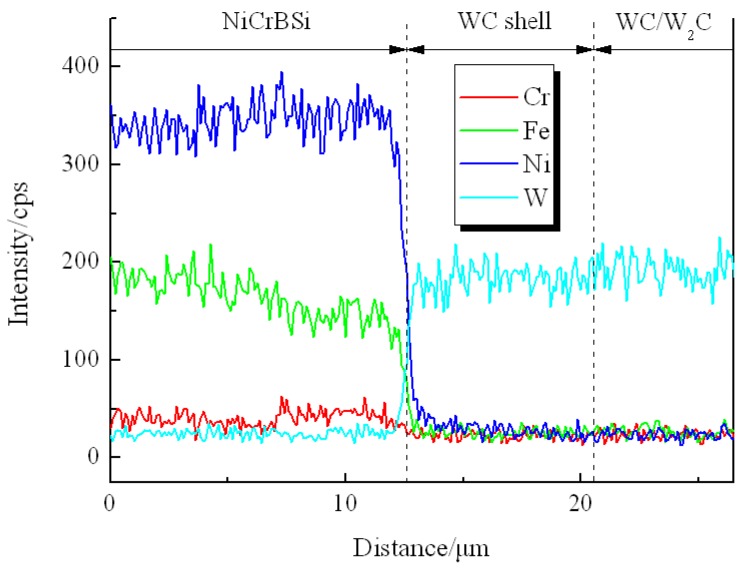
EDS line scan from NiCrBSi to the modified CTC_P_.

The SEM image of the modified CTC_P_-NiCrBSi region in the modified CTC_P_-NiCrBSi/heat resistant steel composite layer is shown in Figure 8. It is evident that the dissolution of the CTC_P_ was largely suppressed. The result of the EDS line scan through the interface between the modified CTC_P_ and NiCrBSi matrix is shown in Figure 9 (scanning line see Figure 8). It is evident that both the outer shell and inner core of the modified CTC_P_ were W-rich with no evidence of nickel. There was a clear boundary between the modified CTC_P_ and the NiCrBSi matrix due to the steep decline in Ni, Fe, and Cr contents and the sharp rise in W content at the interface. A small amount of blocky precipitates (marked as P3) still formed in the matrix. EDS analysis showed that they were also (W, Ni, Cr)-rich carbides which contained more Cr and less W, Ni when compared with that of P1. The formation of P3 might be related to free carbon increasing in the modified CTC_P_ during the carburizing process.

### 3.2. Volume Fraction and Microhardness of the Microstructure Constituent

**Figure 10:**
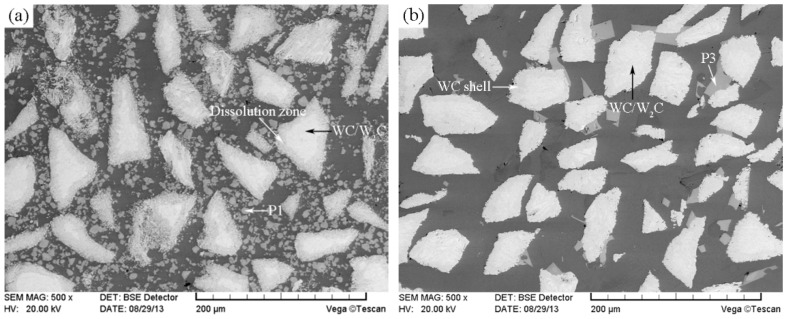
Typical SEM images of (**a**) the unmodified, and (**b**) the modified CTC_P_-NiCrBSi used for quantitative image analysis.

The volume fraction and microhardness of the microstructure constituent in CTC_P_-NiCrBSi region were determined as they are key factors affecting the abrasion resistance of the CTC_P_-NiCrBSi/heat resistant steel composite layers. Figure 10 shows typical SEM images of the unmodified and the modified CTC_P_-NiCrBSi regions, which were used for image analysis. It can be seen that the unmodified CTC_P_ displayed excessive dissolution. The microstructure of the unmodified CTC_P_-NiCrBSi was composed of WC/W_2_C, dissolution zone, precipitate P1, and the NiCrBSi matrix (Figure 10a). The modified CTC_P_ were not affected by dissolution. The microstructure of the modified CTC_P_-NiCrBSi was composed of WC/W_2_C, WC shell, precipitate P3, and the NiCrBSi matrix (Figure 10b). The volume fraction measurement results of the microstructure constituents are listed in Table 1. In the unmodified CTC_P_-NiCrBSi region, the dissolution zone of the CTC_P_ reached 32.7 vol. % and the residual WC/W_2_C eutectic left in the cores was only 7.5 vol. % and the blocky precipitate P1 was up to 18.2 vol. %. In the modified CTC_P_-NiCrBSi region, the WC shell took up 20.1 vol. % and the WC/W_2_C eutectic occupied 19.5 vol. % and the blocky precipitate P3 was only 3.2 vol. %. Compared with the unmodified CTC_P_-NiCrBSi region, an increase of about 12.0 vol. % was achieved in the content of the WC/W_2_C eutectic remaining in the modified CTC_P_-NiCrBSi region.

The microhardness measurement results of the microstructure constituents are listed in Table 2. It can be observed that the mean microhardness of the WC/W_2_C eutectic was over HV2200, which was much higher than that of the dissolution zone (HV1488) and the WC shell (HV1577). The mean microhardness of the precipitate P3 was slightly higher than that of the precipitate P1, which may be related to more Cr element in them.

### 3.3. Interface between CTC_P_-NiCrBSi and Substrate

**Figure 11:**
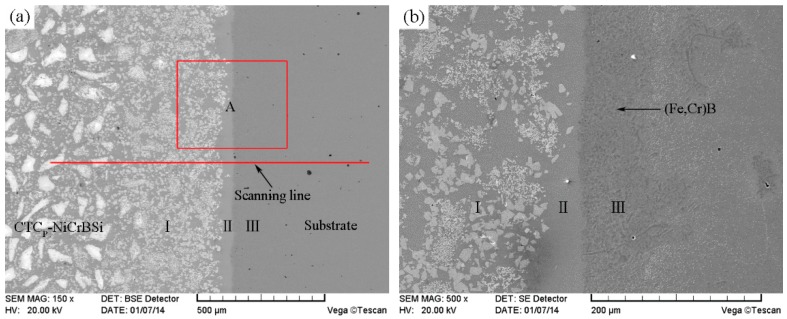
(**a**) SEM image of the interface between unmodified CTC_P_-NiCrBSi and substrate and (**b**) magnified SEM image of the zone “A” in (**a**).

**Figure 12:**
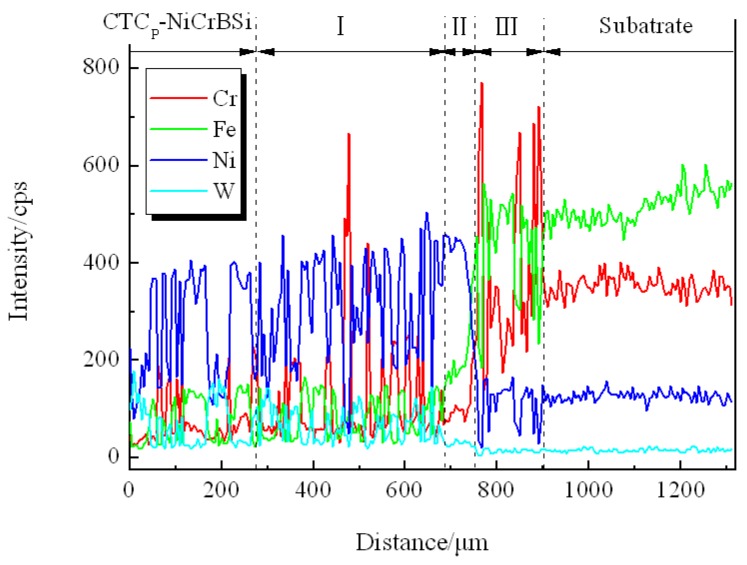
EDS line scan from unmodified CTC_P_-NiCrBSi to substrate.

**Figure 13:**
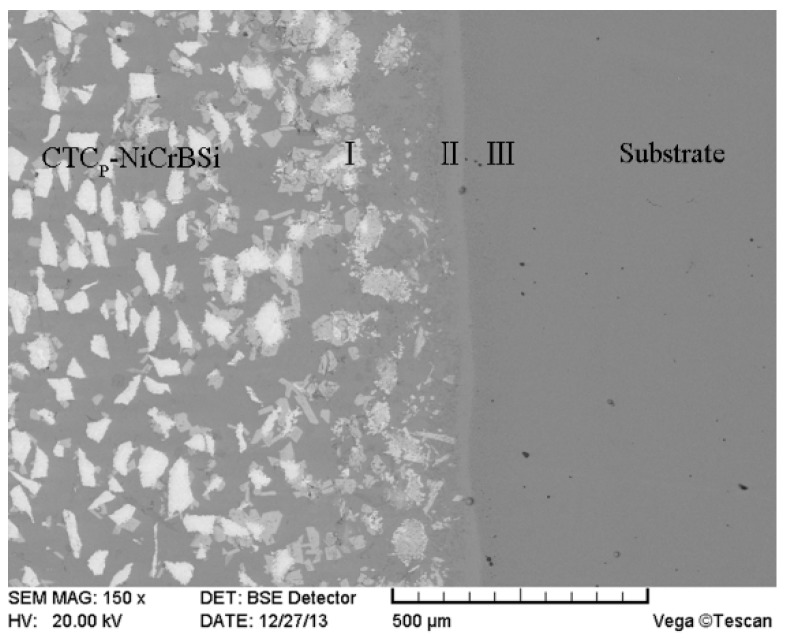
SEM image of the interface between modified CTC_P_-NiCrBSi and substrate.

Figure 11 shows the SEM images of the interface between the unmodified CTC_P_-NiCrBSi and the substrate. It can be seen that a transition layer consisting of three zones formed, which indicates the existence of metallurgical bonding in the interface. From the element distribution profile of the interface in Figure 12 (scanning line see Figure 11a), it is clear that there was a gradual increase in the Fe and Cr contents and a gradual decrease in the W content when the location transited from the unmodified CTC_P_-NiCrBSi to the substrate. This indicates that the dissolution of the substrate and the interdiffusion of elements might take place in the interface. Starting from the unmodified CTC_P_-NiCrBSi and crossing the transition layer, the first zone (labeled as I) was characterized by the unmodified CTC_P_ being completely dissolved where not only the W_2_C, but also the WC in CTC_P_ was dissolved. The excessive dissolution of the unmodified CTC_P_ in this zone might have been caused by the increasing content of Cr and Fe elements which are relatively strong carbide formers. The second zone (labeled as I) was narrower and was constituted by a single phase. EDS analysis results indicated that it was a layer of γ-Ni solid solution rich in Fe, Cr. Therefore, the second zone should belong to the isothermally solidified zone. The third zone (labeled as II) was characterized by many precipitating fine granules (Figure 11b). The fine granules were rich in Cr and Fe elements and their microhardness reached HV900. The fine granules could be (Fe, Cr)B precipitates [17]. So, the third zone should belong to the diffusion affected zone. Figure 13 shows the SEM image of the interface between the modified CTC_P_-NiCrBSi and substrate. It can be seen that the interface also consisted of three zones, but the first zone narrowed down a lot as the modified CTC_P_ had a higher chemical resistance.

**Figure 14:**
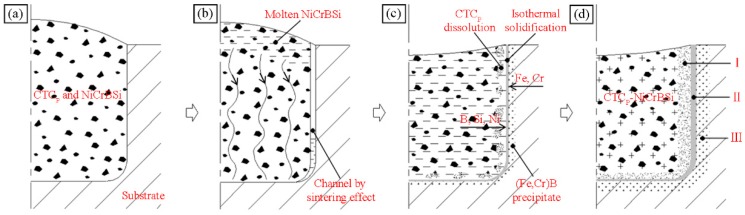
Formation schematic diagram of the interface between CTC_P_-NiCrBSi and substrate: (**a**) before sintering; (**b**) sintering shrinkage and molten NiCrBSi filling channel; (**c**) elements interdiffusion, CTC_P_ dissolution, isothermal solidification, and (Fe,Cr)B precipitation; and (**d**) interface formation.

Figure 14 describes the physical model of the interface microstructure formation mechanism. The mixture of NiCrBSi and CTC_P_ filled the holes of the substrate and compacted under pressure before the vacuum fusion sintering (Figure 14a). During heating to the sintering temperature, the mixture shrank toward the midsection due to the sintering effects, leaving behind a relatively large channel adjoining the faying surface of the substrate. When the NiCrBSi powder deposited at the mouth of the hole became molten, it was drawn into the gap and flowed toward the bottom of the hole preferentially through the capillary passages in the partially sintered mass of mixture. The large channel adjacent to the faying surface of the substrate was filled after the fine capillary passages in the mixture were filled (Figure 14b). In its wake, the molten NiCrBSi dissolved some of the substrate and element interdiffusion occurred in the interface (Figure 14c). In the CTC_P_-NiCrBSi zone near the interface, the Fe and Cr elements resulted from the dissolution of the substrate and the diffusion could promote the dissolution of unmodified CTC_P_, which led to the formation of zone I. As the melting point decreased, elements Si and B diffused outward from the molten NiCrBSi to the substrate, and the melting point of the liquid near the substrate increased, which induced the isothermal solidification resulting in the formation of γ-Ni solid solution layer (zone Ⅱ). Si and B preferred to diffuse along the grain boundaries in the substrate. However, due to the small size of B atoms, the intragranular diffusion of B was essential [18]. As the diffusion continued, the substrate near the interface was enriched with Fe, Cr, and B, so the diffusion affected zone Ⅲ consisting of (Fe,Cr)B precipitates was formed (Figure 14d).

## 4. Wear Behavior

**Figure 15:**
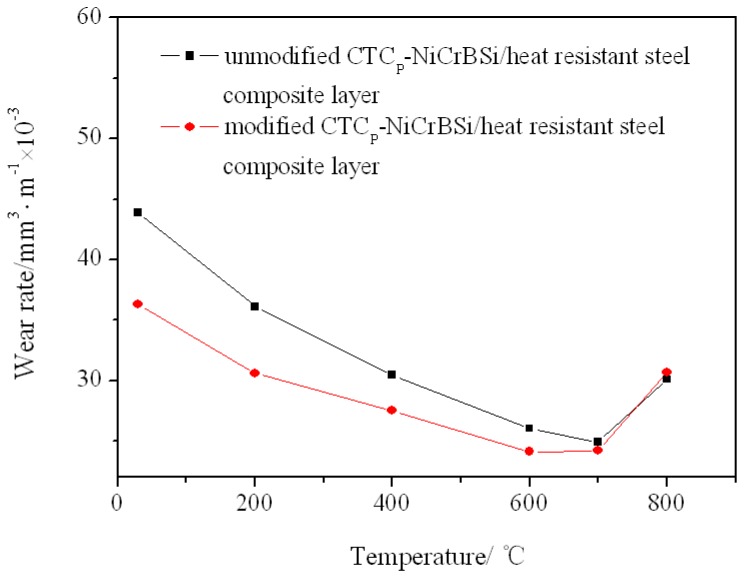
Wear rates of the CTC_P_-NiCrBSi/heat resistant steel composite layers as a function of testing temperature.

The wear rates of the CTC_P_-NiCrBSi/heat resistant steel composite layers investigated at different temperatures are given in Figure 15. It can be seen that the wear rate of the modified CTC_P_-NiCrBSi/heat resistant steel composite layer was lower than that of the unmodified CTC_P_-NiCrBSi/heat resistant steel composite layer between RT and 700 °C, and almost equal above 700 °C. The wear rates of both layers decreased with increasing temperature from RT to 700 °C, and increased above 700 °C. 

**Figure 16:**
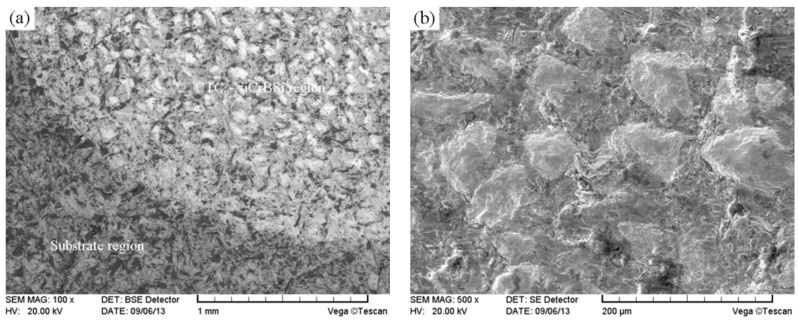
(**a**) Worn surface morphology of the modified CTC_P_-NiCrBSi/heat resistant steel composite layer, and (**b**) magnified worn surface morphology of the CTC_P_-NiCrBSi region at 600 °C.

The worn surfaces were observed after the wear tests. As previously mentioned, there were two kinds of regions distributed in the composite layer, namely the continuous substrate region and the discrete CTC_P_-NiCrBSi regions (Figure 16a). During the abrasion process below 700 °C, the hardness of the substrate region was far lower than that of abrasive and the abrasion loss of the substrate region was larger. In contrast, the abrasion loss of the CTC_P_-NiCrBSi regions was lower because of the existence of high-hardness CTC_P_. The abrasive was unable to scratch the CTC_P_ and the wear was concentrated on the NiCrBSi matrix, which led to the protruded CTC_P_. The protruded CTC_P_ had a protective effect on the NiCrBSi matrix, further on the substrate region (Figure 16b).

**Figure 17:**
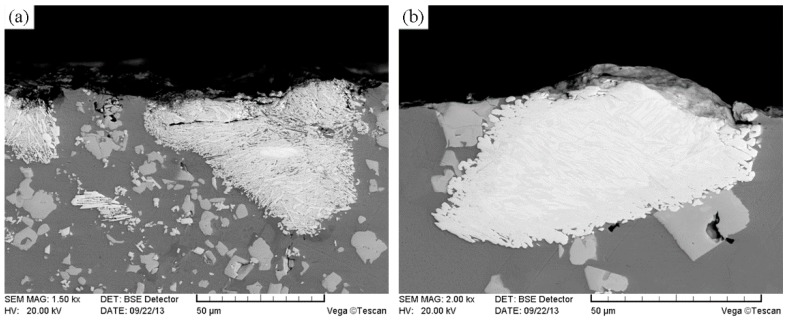
Cross-sections morphologies of (**a**) the unmodified, and (**b**) the modified CTC_P_-NiCrBSi at RT.

Figure 17 shows typical cross-sections of the CTC_P_-NiCrBSi regions in composite layers at RT. Similar features were also observed after tests from 200 °C to 700 °C. It can be seen that the unmodified CTC_P_ did not obviously protrude out of the NiCrBSi matrix as there was less WC/W_2_C left in the core as well as the lower hardness and toughness of the dissolution zone (Figure 17a). Therefore, the protective effect of the unmodified CTC_P_ on the NiCrBSi matrix and substrate region deteriorated. Though a lot of carbides (P1) precipitated in the NiCrBSi matrix because of the dissolution of the CTC_P_, the carbides in size were too small to reduce abrasion. The modified CTC_P_ obviously protruded out of the NiCrBSi matrix and had a better protective effect on the NiCrBSi matrix and substrate region because more fraction of high hardness WC/W_2_C was retained (Figure 17b). Therefore, the wear rate of the modified CTC_P_-NiCrBSi/heat resistant steel composite layer was lower than that of the unmodified CTC_P_-NiCrBSi/heat resistant steel composite layer between RT and 700 °C.

**Figure 18:**
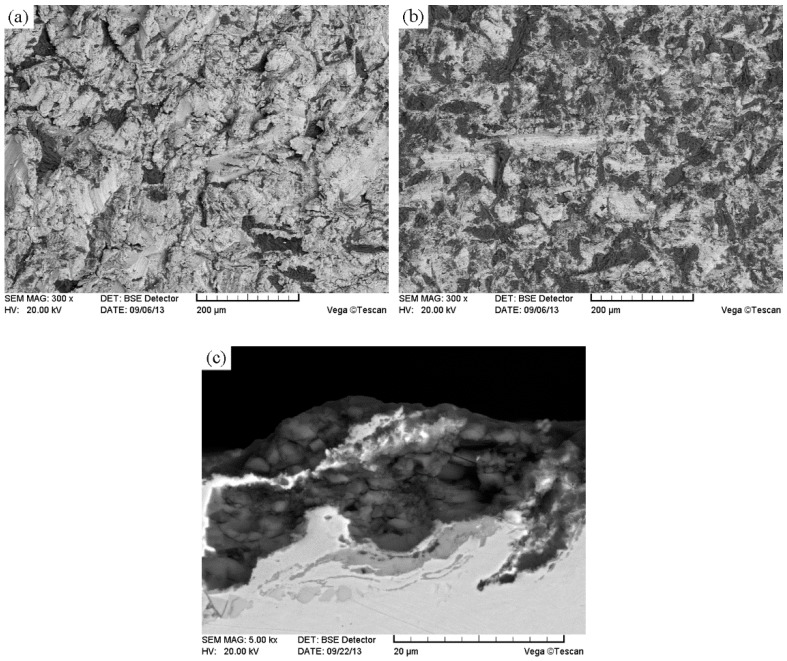
Worn surface morphologies of the substrate regions: (**a**) at RT; (**b**) at 600 °C; and (**c**) cross-section morphology of the substrate region at 600 °C.

Comparing the worn surfaces of substrate regions in the composite layers after tests from RT to 700 °C, it can be seen that the amount of attaching abrasive fragments rose with increasing temperature (Figure 18a,b) and laminated structures consisting of abrasive fragments and wear chips formed on the worn surfaces above 400 °C (Figure 18c). These observed features were in agreement with other studies [19,20]. The higher the temperature, the softer the substrate and the more abrasive fragments penetrated the worn surfaces. The formation of the laminated structures could be due to metal chips formed by cutting, which were then compacted back on to the disc [21]. In the CTC_P_-NiCrBSi regions, only a few abrasive fragments and laminated structures formed in the NiCrBSi areas between the protruding CTC_P_, which may be due to a higher hot hardness of the NiCrBSi matrix. The abrasive fragments and laminated structures act like hard phases and protect the surfaces against wear. In addition, when the fragments are loose, they may act as a fluidized bed that also reduces friction and wear [22]. Therefore, the wear rates of both composite layers decreased with increasing temperature from RT to 700 °C.

**Figure 19:**
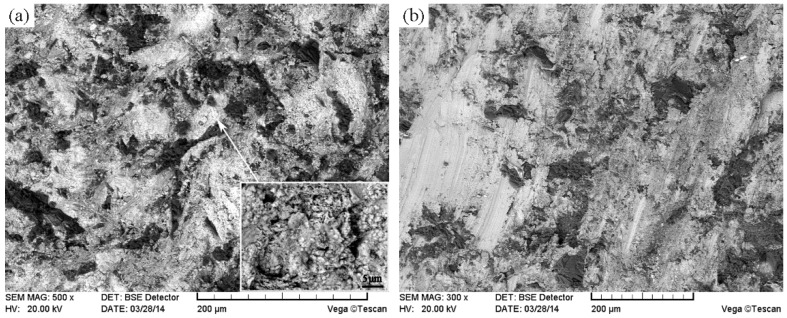
Worn surface morphologies of the modified CTC_P_-NiCrBSi/heat resistant steel composite layer at 800 °C: (**a**) CTC_P_-NiCrBSi region, and (**b**) substrate region.

Typical worn surfaces of the modified CTC_P_-NiCrBSi/heat resistant steel composite layer at 800 °C are illustrated in Figure 19. Similar features were also observed for the unmodified CTC_P_-NiCrBSi/heat resistant steel composite layer. It can be seen that the white flakes were generated due to CTC_P_ fracture. These wear debris were randomly distributed on the worn surface. The CTC_P_ in SEM high magnification mode showed cracks and pores (Figure 19a). EDS analysis showed that the oxygen concentrated on the surfaces of the CTC_P_ was up to 68.73 at. %, which indicates that excessive oxidation of CTC_P_ occurred. In addition, massive grooves formed on the worn surface of the substrate region (Figure 19b). Below 600 °C, the oxidation of CTC_P_ during the abrasion process in air was moderate [23] so they provided good wear protection. While above 600 °C, the oxidation of CTC_P_ increased rapidly with temperature. The porous oxides offered no resistance to abrasion in air. Additionally, as the depth of the grooves produced by the abrasive particles was larger than the thickness of the laminated structures and the sizes of the abrasive fragments, they were moved from the surface. Therefore, above 700 °C, both the degradation of the CTC_P_ by oxidation and the massive ploughing effects resulting from the softening of the matrix were responsible for the increase of wear rates in Figure 15.

## 5. Conclusions

1. A CTC_P_-NiCrBSi/heat resistant steel composite layer with architecture similar to concrete was designed for the toughening layer and fabricated by vacuum fusion sintering, which contained no obvious defects such as cracks and pores.

2. The poor chemical resistance of W_2_C and the interdiffusion of elements were responsible for the dissolution of unmodified CTC_P_ in the molten NiCrBSi alloy. The W_2_C in CTC_P_ reacted with Ni and Cr diffusing from molten NiCrBSi to form (W,Ni)-rich carbides, while WC remained mostly intact. Ni, Cr, and Fe in NiCrBSi reacted with W and C diffusing from CTC_P_ to form (W,Ni,Cr)-rich carbides. These (W,Ni,Cr)-rich carbides precipitated from the melt during cooling.

3. The carburized WC outer shell of the modified CTC_P_ could effectively resist the dissolution during vacuum fusion sintering. Compared with unmodified CTC_P_-NiCrBSi, an increase of about 12.0 vol. % in the content of WC/W_2_C eutectic remaining in the modified CTC_P_-NiCrBSi was achieved.

4. The wear rate of the modified CTC_P_-NiCrBSi/heat resistant steel composite layer was obviously lower than that of the unmodified CTC_P_-NiCrBSi/heat resistant steel composite layer between RT and 700 °C, and almost equal above 700 °C. The wear rates of both composite layers decreased with increasing temperature from RT to 700 °C and increased above 700 °C.

## Figures and Tables

**Table 1:** Volume fraction of the microstructure constituent in CTC_P_-NiCrBSi.

CTC_P_-NiCrBSi Regions	Constituent Content (vol. %)					
	WC/W_2_C	Dissolution Zone	WC Shell	P1	P3	NiCrBSi Matrix
Unmodified	7.5	32.7	-	18.2	-	balance
Modified	19.5	-	20.1	-	3.2	balance

**Table 2:** Microhardness of the microstructure constituent in CTC_P_-NiCrBSi.

CTC_P_-NiCrBSi Regions	Constituent Microhardness, HV				
	WC/W_2_C	Dissolution Zone	WC Shell	P1	P3
Unmodified	2256	1488	-	1084	-
Modified	2231	-	1577	-	1262
